# Application of microfluidic systems in modelling impacts of environmental structure on stress-sensing by individual microbial cells

**DOI:** 10.1016/j.csbj.2021.11.039

**Published:** 2021-12-01

**Authors:** Harry J. Harvey, Mykyta V. Chubynsky, James E. Sprittles, Leslie M. Shor, Sacha J. Mooney, Ricky D. Wildman, Simon V. Avery

**Affiliations:** a School of Life Sciences, University of Nottingham, Nottingham, UK; b Mathematics Institute, University of Warwick, Coventry, UK; c Department of Chemical and Biomolecular Engineering, University of Connecticut, USA; d School of Biosciences, University of Nottingham, Nottingham, UK; e Faculty of Engineering, University of Nottingham, Nottingham, UK

**Keywords:** Structured microenvironments, Environmental heterogeneity, Fluid flow modelling, *Saccharomyces cerevisiae*, Stress response, Copper stress

## Abstract

Environmental structure describes physical structure that can determine heterogenous spatial distribution of biotic and abiotic (nutrients, stressors etc.) components of a microorganism’s microenvironment. This study investigated the impact of micrometre-scale structure on microbial stress sensing, using yeast cells exposed to copper in microfluidic devices comprising either complex soil-like architectures or simplified environmental structures. In the soil micromodels, the responses of individual cells to inflowing medium supplemented with high copper (using cells expressing a copper-responsive *pCUP1*-reporter fusion) could be described neither by spatial metrics developed to quantify proximity to environmental structures and surrounding space, nor by computational modelling of fluid flow in the systems. In contrast, the proximities of cells to structures did correlate with their responses to elevated copper in microfluidic chambers that contained simplified environmental structure. Here, cells within more open spaces showed the stronger responses to the copper-supplemented inflow. These insights highlight not only the importance of structure for microbial responses to their chemical environment, but also how predictive modelling of these interactions can depend on complexity of the system, even when deploying controlled laboratory conditions and microfluidics.

## Graphical abstract

**Figure:**
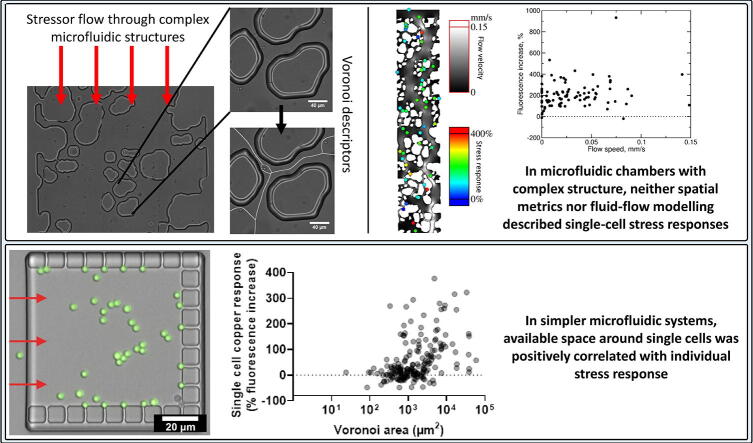

## 1 Introduction

Microorganisms such as bacteria and fungi are subject to temporal and spatial variation in the abiotic (nutrients, oxygen, temperature etc.) and biotic factors that shape their microenvironments, metabolism and proliferation [12], [20]. A key factor determining such physicochemical gradients in a cell’s (micro)environment is environmental structure, here in reference to physical structures that may promote environmental heterogeneity. The soil habitat is one example of a structured environment, with soil components forming complex networks of both connected and isolated pores that can house diverse microbial life [15]. Other examples of porous microenvironments range from virtually all natural microbial habitats to biomedical devices [8] and hygenic surfaces [30].

The importance of structured microenvironments, and ways of studying them in laboratory systems, has recently attracted increasing focus [1], [24], [10], [13]. For example, it has been demonstrated that bacterial biofilm formation can enhance water retention and reduce water evaporation rates within soil-micromodel microfluidic devices [24] and that the biofilm forming ability of some cells can facilitate the persistence of non-biofilm forming organisms within structured environments [18]. However, there are very few studies of how micrometre-scale environmental structure impacts the sensing (exposure and response) by microorganisms of environmental stimuli. This is important considering that microorganisms residing in structured environments such as soil are vital for biogeochemical cycling, but are subject to stressors such as toxic pollutants (e.g., metals and microplastics) [29], [23] and other environmental perturbations such as temperature- and rainfall-fluctuations, mechanical disturbance, etc. [31]. Therefore, understanding how environmental micro-structure influences the responses of microbial communities to perturbation will enable deeper predictive understanding of the impacts of such perturbation on essential microbial services.

Examining the interaction between microorganism and abiotic factors in structured environments at small scales is challenging [3], [10]. However, the application of microfluidic technology (which allows precise manipulation of fluid flow at the microlitre scale and below) in systems with incorporated structured elements offers potential for examining this interaction, as microfluidic devices enable the precise control of a cell’s microenvironment alongside convenience as a platform for single cell imaging and tracking [6], [5], [18], [10]. Moreover, custom microfluidic devices have been developed with incorporated soil-relevant structures, termed soil micromodels [6]. These offer an added level of environmental complexity to that achievable by introduction of simple structures such as inert particles or barriers into otherwise homogeneous chambers of microfluidic devices. The power of computational fluid-flow modelling enables characterisation of resultant perturbations to fluid perfusion through these structured systems. In this study, these tools were combined to describe the effects of environmental structure in microfluidic systems on the sensing of elevated environmental-stressor levels by single yeast cells. The yeast *Saccharomyces cerevisiae* provided an especially suitable model as its molecular responses to chemical stress – including the exemplar chosen for this study, copper, an important pollutant from mining and other industrial effluents [29]– and suitable genetic tools are very well characterized [17], [26].

## 2 Methods

### 2.1 Yeast strains and culture conditions

*Saccharomyces cerevisiae* SVY14 *HO:*:*pCUP1-yEGFP* (in the background MATa *leu2-3*, *112 ura3-52 trp1-289*) was constructed previously [17]. Yeasts were maintained and grown in YNB medium [0.69% yeast-nitrogen base without amino acids (Formedium), 2% (w/v) D-glucose], supplemented as required with amino acids or nucleobases to complement auxotrophies (as listed above). Where necessary, media were solidified with 2% (w/v) agar (Sigma-Aldrich, St. Louis, MO). For experiments, single colonies were used to inoculate 10 ml of medium in 50 ml Erlenmeyer flasks and incubated overnight at 30 °C with orbital shaking (New Brunswick Scientific) at 120 rev. min^−1^. To produce exponential phase cells for experimental purposes, overnight cultures were diluted to OD_600_ ∼ 0.5 and incubated as above until cells reached an OD_600_ ∼ 1.5.

### 2.2 Determination of cellular GFP with flow cytometry

Single-cell fluorescence from expression of GFP was determined for samples (500 µl) of exponential phase cells from *S. cerevisiae* SVY14 cultures at OD_600_ ∼ 0.5 in YNB medium following incubation for either one or two hours with added copper sulfate (CuSO_4_) at specified concentrations. After copper treatment, cells were harvested by centrifugation at 4,500 *g* for 5 min, the supernatant removed, and cells washed twice in phosphate buffered saline (PBS) (137 mM sodium chloride, 2.7 mM potassium chloride, 11.9 mM phosphate buffer) at room temperature. Cellular GFP fluorescence was determined for 10^6^ cells (events) per sample by flow cytometry, with a FACSCanto A (BD Biosciences) instrument. Laser excitation was at 488 nm and emission was collected through a FITC 530/330 nm filter. Events were gated by median forward scatter and side scatter to exclude doublets and debris. Median fluorescence of gated cells was then calculated using Flowing Software V2.5 (Turku Bioscience).

### 2.3 Soil micromodel experiments

**Fig. 1:**
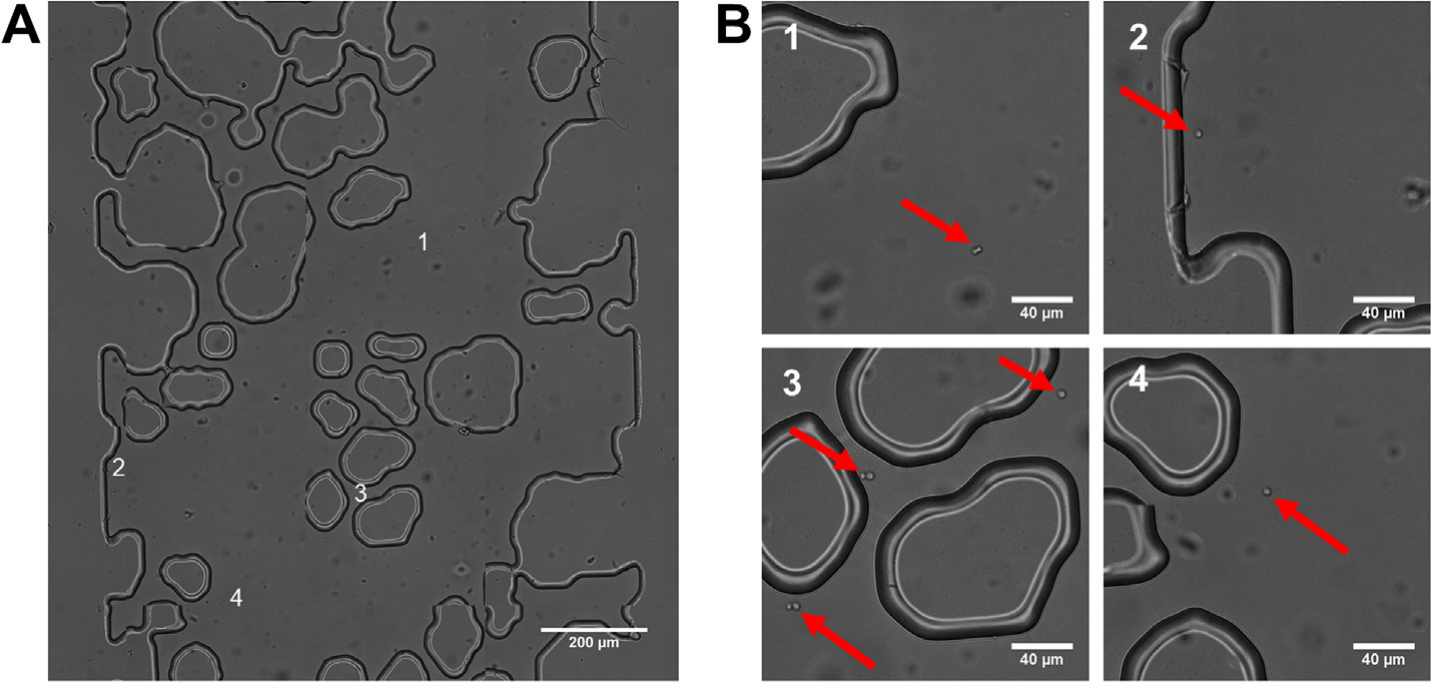
**Representative microscopic images ofsoil micromodels.** Soil-micromodel microfluidics devices contained solid PDMS structures within a channel 1 mm in diameter and 10 mm in length, with the direction of fluid flow from top to bottom of the images; approximately one fifth of the channel length is presented in (A). Cells are distributed randomly through the open pore space, with examples of cell location highlighted at positions 1–4 in (A) and magnified in the corresponding close-ups presented in (B), where cells are indicated by arrows.

The soil micromodels described here are microfluidic devices consisting of a simulated soil structure moulded in polydimethylsiloxane (PDMS) polymer and plasma bonded onto a glass substrate, prepared as described previously [24]. Micromodels were sterilised before experiments with a 30 W ultraviolet lamp source (Philips) at 60 cm distance, 20 min. The inner surfaces of sterile micromodels were coated with 2 mg ml^−1^ concanavalin A (ConA) (Sigma-Aldrich) by introducing ConA solution through one of the inlets located at either end of the model and flowing through until the model was saturated, then incubated overnight. This was to promote subsequent cell adhesion to the glass floor of the device. Micromodels were then flushed with filtered (pore diameter, 0.22 µm) YNB medium to remove excess ConA solution and then inoculated with cells suspended at 750 cells µl^−1^ in YNB, by flowing the suspension into the model at a rate of 10 µl h^−1^ until cells were present throughout the model, resulting in ∼ 100 cells per micromodel channel. Flow was controlled using a 20 ml syringe connected to the inlet of the microfluidic device, with the syringe mounted on a NE-500 syringe pump (New Era Pump Systems, Inc.) set to apply a force on the syringe plunger flange as appropriate for the desired flow rate; the syringe pump was controlled using SyringePumpPro software (SyringePumpPro). Devices were then mounted onto an inverted microscope stage maintained at 30 °C. Cells were allowed to settle to the glass floor of the device for 20 min (Fig. 1) before a flow of YNB medium was introduced at 2 µl hr^-1^ for 20 min to flush out non-adherent cells.

Before copper sulfate was introduced, micromodel channels were imaged in brightfield transmitted light and GFP emission wavelengths, to record the micromodel structure and baseline fluorescence values for individual cells (image acquisition parameters are detailed in 2.6). For the copper stress, the syringe and tubing were replaced with a syringe and tubing containing YNB supplemented with 200 µM CuSO_4_. This copper supplemented medium was then introduced to the model at the same flow rate as above, before imaging again after 1 h.

### 2.4 Flow simulation within soil micromodels

#### 2.4.1 Copper ion movement: General considerations

Fluid flow simulations were conducted to model movement of dissolved copper through the soil micromodel system. In the system, copper ions both are advected with the flow of the medium and they diffuse, and both of these effects are important. In a uniform flow with speed v, diffusion dominates at distances r≲D/v, where D is the diffusion coefficient of the ions. Ions are brought into the neighbourhood of the cell of size ∼*r* by advection and then diffuse towards the cell. The ion flux towards the cell is expected to be approximately proportional to the average flow speed within distance *r* of the cell. Using $D\approx 7\times 10^{-10}m^{2}/s$ for Cu(II) ions in water [19], [32] and a typical flow speed in the pores ∼10^−4^ m/s (see below) gives an approximation of *r* of a few microns, comparable both to the yeast cell size and the typical pore size in the soil micromodels, although usually smaller than the latter. Note that all cells adhere to the floor of the channel, and many also to the pore walls where the flow speed (assuming no slip at the walls) approaches zero. Therefore, it is important to consider flow in a neighbourhood of the cell rather than just the speed at its location.

#### 2.4.2 Modelling assumptions

To model the fluid flow through soil micromodels structures three main assumptions were made. First, that the flowing medium is a Newtonian fluid, i.e., its viscosity is constant and does not depend on the shear rate. Although some shear thinning of the medium was evident, the viscosity changed by a factor of less than two when the shear rate changed by a factor of ten (from 10 to 100 s^−1^). Therefore, neglecting this effect appeared reasonable as it should not change the results qualitatively. Second, the flow obeys the Stokes flow equation, i.e., inertia is neglected. Indeed, even assuming that the entire flow (flow rate Φ = 5.56 × 10^−13^ m^3^/s) is concentrated in a single pore of width wand height equal to the structure height, h=32μm, and using w as the characteristic length scale, gives the estimate of the Reynolds number $Re∼\rho \Phi /\left(\mu h\right)∼10^{-2}\ll 1$, where the density $\rho ∼10^{3}\;kg/m^{3}$ and the viscosity $\mu ∼10^{-3}\;Pa\;s$. Third, the medium is an incompressible fluid. Then the flow obeys the Stokes equation

(1) $$-\nabla p+\mu \nabla^{2}v=0$$

and the continuity equation

(2) ∇·v=0

with velocity v. The density ρ is not present in these equations as inertia is neglected.

#### 2.4.3 A two-dimensional approximation of fluid flow

The coordinate system of the micromodels was defined such that the *z* coordinate axis is perpendicular to the floor, the ceiling ‘covers’ the structure and the system spans the *z* range from 0 to h. Although the structure is two-dimensional, the flow is not strictly 2D because the system has a finite height, neither very small nor very large compared to pore widths, so that flow varies in the *z*-direction. Initially the limits of very large and very small heights were considered and then it was determined how the intermediate case can be treated. First, if the height h is much larger than any of the features of the structure, e.g., pore widths (referred to as the “thick limit” for brevity), then the covers have an effect only in a small fraction of the volume close to them. The flow is nearly horizontal in all locations, with the flow speed only approaching zero very close to the covers. Neglecting these small regions near the covers, the velocity becomes strictly 2D,

(3) v=v(x,y)

with no z dependence and also no z component. Then in Eqs. (1), (2) the ∇ operator is simply replaced by the 2D one $\nabla_{⊥}=\left(\partial /\partial x,\partial /\partial y\right)$, giving the 2D Stokes equations

(4) $$\begin{array}{c} -\nabla_{⊥}p+\mu \nabla_{⊥}^{2}v=0 \end{array},$$

(5) $$\nabla_{⊥}\cdot v=0.$$

The opposite limit, when the height is much smaller than any other features within the micromodel (the “thin limit”), gives the Hele-Shaw cell approximation, where the velocity gradient is much larger in the z direction than in the other two directions. Then Eq. (2) becomes $\partial v_{z}/\partial z=0$, which with impermeability at the top and bottom must give

(6) $$v_{z}=0$$

The z component of Eq. (1) then gives ∂p/∂z=0, so

(7) p=p(x,y)

and in the xy plane giving

(8) $$-\nabla_{⊥}p+\mu \frac{\partial^{2}v}{\partial z^{2}}=0$$

which can be solved for v and, together with Eq. (2), gives the following equations:

(9) $$\begin{array}{cc} v & =-\frac{1}{2\mu }\left(\nabla_{⊥}p\right)z\left(h-z\right) \end{array},$$

(10) $$\nabla_{⊥}\cdot v=0.$$

Averaging Eq. (9) in the z direction gives the averaged velocity

(11) $$\overset{-}{v}\left(x,y\right)=-\frac{h^{2}}{12\mu }\nabla_{⊥}p$$

and Eq.(10) gives

(12) $$\nabla_{⊥}\cdot \overset{-}{v}=0$$

Substituting Eq. (11) into Eq.(12) gives

(13) $$\nabla_{⊥}^{2}p=0$$

The equations are equivalent to those for irrotational (potential) flow, which is the flow encountered for zero viscosity liquid in the system of arbitrary thickness, where solution methods are well established. For soil micromodels, neither of the above limits apply across the whole system, as there are pores both wider and narrower than h. Because of this, the equations were combined in the two limits, in effect, interpolating between them; the resulting system of equations is then expected to be approximately valid even when the system contains both wide and narrow pores, as well as those of intermediate width.

It is noted that in the thick limit the velocity is nearly independent of z, and v can be replaced by $\overset{-}{v}$ in Eqs. (4), (5). Then the form of the continuity equation is the same in both limits [cf. Eqs. (5), (10)] and it should be valid in the intermediate case as well. This is an exact result, not an approximation, as it simply follows from mass conservation. Furthermore, in both limits p=p(x,y) and it is assumed that this is still valid in the intermediate case. Equations (4), (11) are different and need to be combined. They can be rewritten as

(14) $$\nabla_{⊥}p=\mu \nabla_{⊥}^{2}\overset{-}{v}\text{(thick limit)}$$

and

(15) $$\nabla_{⊥}p=-\frac{12\mu }{h^{2}}\overset{-}{v}\text{(thin limit)}$$

respectively. Adding up the right-hand sides gives

(16) $$\nabla_{⊥}p=\mu \left(\nabla_{⊥}^{2}\overset{-}{v}-\frac{12}{h^{2}}\overset{-}{v}\right)$$

Conveniently, this equation reduces to Eq. (14) in the thick limit and to Eq. (15) in the thin limit, thus interpolating between them, as desired. Eqs. (16), (12) constitute a system that needs to be solved numerically.

As usual for the Stokes equations, the system of equations (16), (12) requires boundary conditions for both components of the velocity on solid surfaces (pore walls). It is assumed that there is no slip, thus both velocity components are zero. Note that thin-limit equations (11)-(12) require only one boundary condition, for the normal velocity. While in Eqs. (16), (12) the tangential component is zero at the walls, for small h it rises rapidly to a nonzero value in a thin boundary layer whose thickness tends to zero as h→0, consistent with the thin-limit equations. At the entrance to and exit from the micromodels, boundary conditions must be consistent with the flow rate. For analysis, a fixed normal flow speed $v_{0}$ was imposed at the inlet and a fixed pressure at the outlet (using other boundary conditions, for example, imposing an average normal velocity equal to $v_{0}$ and no tangential velocity, had no visible effect on the results). The inflow speed $v_{0}$ was calculated by using the flow rate Φ and the cross section of the channel (height h as given above and width =0.974mm, which gives $v_{0}=1.78\times 10^{-5}\;m/s$).

### 2.5 Experiments within CellASIC microfluidic devices

Commercially available CellASIC ONIX pad trap plates (Sigma-Aldrich) were used to create simple micrometre-scale structures for stress response experiments. The plates comprise four chambers, each chamber containing 104 cell ‘traps’ (each trap measuring 100 × 100 µm) and consisting of a perimeter of pillars to help retain cells while permitting fluid flow through the traps (Supp. Fig. 1**D**). A 4 µm ceiling-height within traps is used to help to stop further movement of yeast cells (∼4–5 µm diameter); the ceiling height surrounding traps is ∼ 20 µm. The CellASIC system is driven by a constant pressure (unlike the flow-driven soil micromodels described above), where fluids to be introduced to the micromodel chambers are held within 500 µl solution inlets and a valve system used to regulate pressure and temperature within the plates (Supp. Fig. 1**A,C**). Pressure is applied to each solution inlet individually to introduce flow of a particular fluid into the microfluidic chamber. For experiments, the plate temperature was maintained at 30 °C. Plate chambers were inoculated either with cells of *S. cerevisiae* SVY14 at OD_600_ ∼ 0.1 (or OD_600_ ∼ 0.3 for assay of flow-rate effect on stress response), or cells at the same concentration mixed with 4 µm TetraSpeck microspheres (Invitrogen) at either 1.26 × 10^7^ or 6.3 × 10^6^ sphere particles ml^−1^, suspended in YNB medium. Cells and/or microspheres were introduced to the chambers by flowing these mixtures into the model at 8 psi in three 10 s bursts (or one burst for assay of flow-rate effect on stress response), which resulted in chambers containing ∼ 1 yeast cell per trap and an average of either 0, 16, or 39 microspheres per trap, depending on the microsphere inoculum, to create structured chambers with different structure densities (Supp. Fig. 1**D**). To help distribute microspheres within each trap and reduce aggregation at the trap perimeter, the flow direction was alternated at 5 psi in short bursts (3–4 s). This “shuffled” microspheres away from the trap perimeter, reducing the number of microspheres that might impede fluid flow into or out of the traps.

After introduction and distribution of cells and spheres, flow of YNB medium was introduced across all three chambers of a plate at 2 psi for 20 min before imaging in brightfield and at GFP excitation/emission wavelengths, as described in 2.6. Subsequently the system was flushed with YNB supplemented with 200 µM CuSO_4_ at 8 psi for 10 s, then flow of the same fluid was reduced to 2 psi (1 psi was also assayed for effect of flow-rate on stress response) and continued for 1 hr before cells within chambers were imaged again after the copper stress.

For experiments measuring the impact on fluid flow of microspheres present in the CellASIC cell traps, the fluorescent dye rhodamine 6G (Sigma-Aldrich) at 250 µM was introduced into either empty or microsphere supplemented traps, with fluorescence images taken at intervals using the same imaging parameters as for GFP measurements (2.6). To analyse the data with Fiji v1.51w (see 2.6), a straight-line section was drawn from the trap opening to the back of the trap, and the mean fluorescence intensity along this line measured at every 100 ms time interval using the “Plot profile” function, enabling a representative measurement of dye flow across the whole trap.

### 2.6 Microscopy and imaging

All microscopy and imaging was conducted at the School of Life Sciences Imaging (SLIM) Centre, University of Nottingham. Soil micromodels were examined with a DeltaVision Elite Microscope (Applied Precision/GE Healthcare) equipped with a 20x, 0.85NA objective. Fluorescence excitation was at 475 nm (bandwidth 28 nm) and emission measured at 525 nm (bandwidth 50 nm). Images were captured using a CoolSnap HQ2 CCD camera (Photometrics) at 60 ms exposure. Brightfield transmitted light images were acquired with 10 ms exposure. CellASIC microfluidics plates were examined using a Zeiss Exciter Widefield microscope equipped with a 20x, 0.50NA objective. Fluorescence excitation was at 470 nm (bandwidth 40 nm) and emission recorded at 525 nm (bandwidth 50 nm). Fluorescence and brightfield images were captured using a Retiga R1 CCD camera at 60 and 10 ms exposure times, respectively. For both systems, the cell chambers were imaged over multiple fields of view using a motorised stage for multi-point visiting controlled using Micro-Manager software V1.4 software, applying a 10% image overlap between panels to allow image stitching post acquisition.

#### 2.6.1 Image analysis

Image analysis was performed using Fiji v1.51w software [25]. Images from multi-point acquisition were assembled into one larger image using the Grid/Collection plugin V1.2 [22]. Voronoi areas and greyscale distance maps (described in 3.2) were calculated using Fiji V.151w built-in plugins. Yeast cells were identified and selected manually, and fluorescence values calculated as mean intensity of pixels within each cell. The mean background fluorescence signal was subtracted from mean intensity values of all cells prior to calculating fluorescence increases. For all microfluidics experiments, the same total area for each yeast cell was measured at each timepoint, and cells which appeared to be doubling (determined visually) during the experiment were excluded from analysis.

#### 2.6.2 Single cell spatial analysis

Voronoi tessellations, used as a measure for the space surrounding individual cells, were generated in a semi-automated process in Fiji v1.51w using the “Voronoi” plugin. For greyscale distance mapping within Voronoi areas, each pixel of a cell’s Voronoi area was weighted linearly according to its distance from the cell centre, starting at a value of 1. Thus, a pixel adjacent to the cell centre would be assigned a value of 1 and a cell 200 pixels from the cell centre assigned a value of 201. Voronoi measurements and the greyscale mapping were collectively termed spatial metrics.

## 3 Results

### 3.1 Using fluorescent reporter-protein expression as a proxy for cellular exposure to copper

**Fig. 2:**
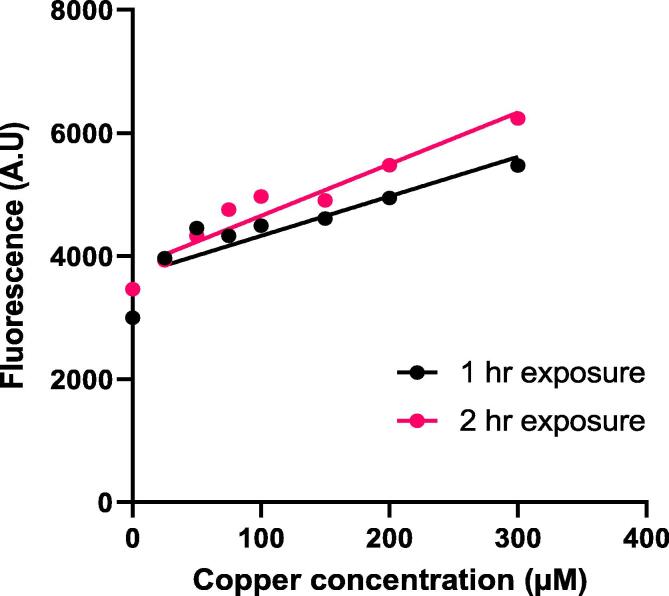
**Correlation of *pCUP1*-GFP expression with supplied copper concentration.** Fluorescence of single cells expressing GFP under control of the *CUP1* promoter after incubation in YNB medium with a range of supplemented copper sulfate concentrations for either 1 or 2 h. Cell fluorescence was measured by flow cytometry. Each point represents median cell fluorescence measured across 100,000 cells. The average coefficient of variation (CV) was 63.8%. Data for the no-copper controls were excluded from the linear regression plot as these deviated from linearity.

In order to investigate the relationships between single-cell exposure to elevated copper and parameters of environmental structure, first the range over which the *pCUP1*-GFP reporter of copper stress could be used to report reliably on copper sensing by the cells was assayed. The yeast *CUP1* gene encodes its major copper metallothionein and is strongly inducible by high copper levels [17]. *S. cerevisiae* SVY14 expressing the *pCUP1*-GFP showed a linear, positive correlation between the concentration of copper supplied to cells and cellular fluorescence, after either 1 hr or 2 hr incubation in flask cultures over the final, sub-inhibitory concentration range of 25 to 300 µM of added copper sulfate (at 1 hr R^2^ = 0.939, p < 0.001; at 2 hr R^2^ = 0.942, p < 0.001). Including the no-copper control in this range gave a deviation from linearity, as it was noted that the fluorescence increase between 0 and 25 µM was larger than in subsequent increments of supplied copper concentration. The detected response of cells was greater at 2 hr exposure across all concentrations (Fig. 2).

### 3.2 Use of defined “spatial metrics” as measures of a cell’s local environmental structure

**Fig. 3:**
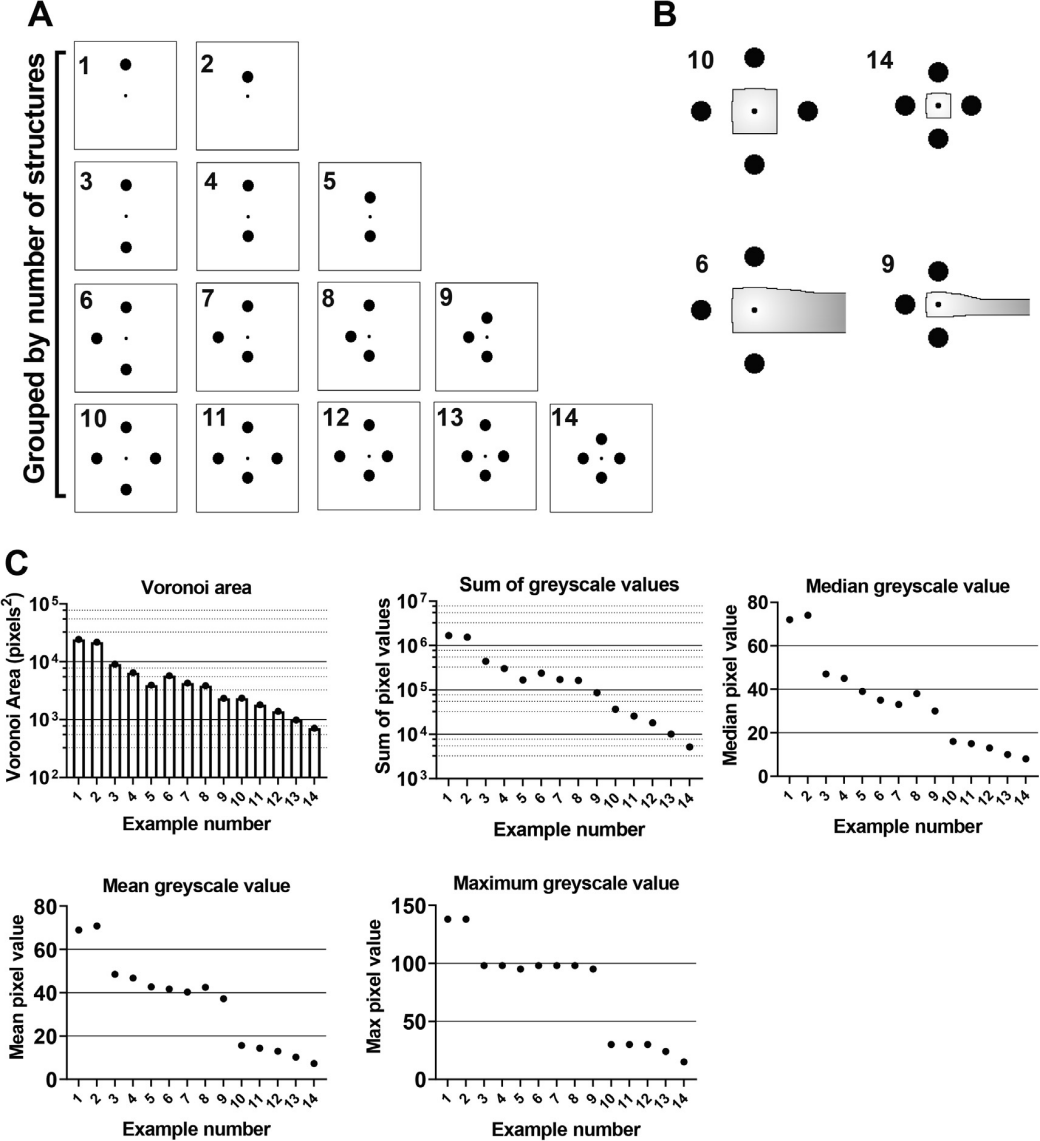
**Exploring spatial metrics for describing the spatial relationships between points (cells) and objects.** (A) A systematic array of central points (smaller circle, representing a microbial cell) with an increasing number of objects (larger circles, representing a simple environmental structure) either one or two object diameters away from the central point. (B) Selected illustrations of Voronoi tessellation (black outline), around a central point, dividing the open space between objects. Each pixel within the tessellation is given a value corresponding to its distance from the central point, illustrated here as greyscale ranging from 0 (white) to 255 (black). The values of all measured parameters for each numbered example image in (A) are presented in panel (C).

Before progressing with copper response experiments, a set of parameters were considered for suitability as descriptors of the local physical surroundings of individual cells. First, Voronoi areas were determined as a descriptor of the proximity of cells to surrounding objects. Voronoi areas are defined by tessellations that separate the open space between points, such that any space within a point’s Voronoi area is closer to that point than any other object. A series of mock images were produced, starting with a single central point (representing a microbial cell) and object (representing an environmental structure), followed by the systematic addition of objects at distances equal to one or two object-diameters from the central cell (Fig. 3**A**). This produced an array of spatial configurations for analysis. Voronoi areas (illustrated in Fig. 3**B)** were then calculated for each cell relative to its surrounding objects (see Methods section 2.6.1). Subsequently each pixel within a Voronoi area was assigned a numerical value corresponding to its distance from the cell, represented as a greyscale distance map (with values from 0 to 255 encompassing white to black, respectively) (Fig. 3**B**). This allowed Voronoi areas to be weighted in a way that reflected differing shapes, as Voronoi areas encompassing larger distances between the cell and area-perimeter included larger numerical values. Plotting cells’ Voronoi areas, or other parameters from the greyscale distance maps, in order of the inferred, relative complexity of the different configurations that were trialled showed the anticipated trend. That is, either an increased number of objects around a cell or a reduced distance between objects and cell, reduced the Voronoi area and greyscale distance values, reflecting reductions in open space around the cell (Fig. 3**C**). It should be noted that some measures, such as median greyscale value, showed this general trend but also showed deviation from the trend as the object number was increased.

### 3.3 Responses of individual yeast cells to copper in soil micromodels and application of spatial metrics

**Fig. 4:**
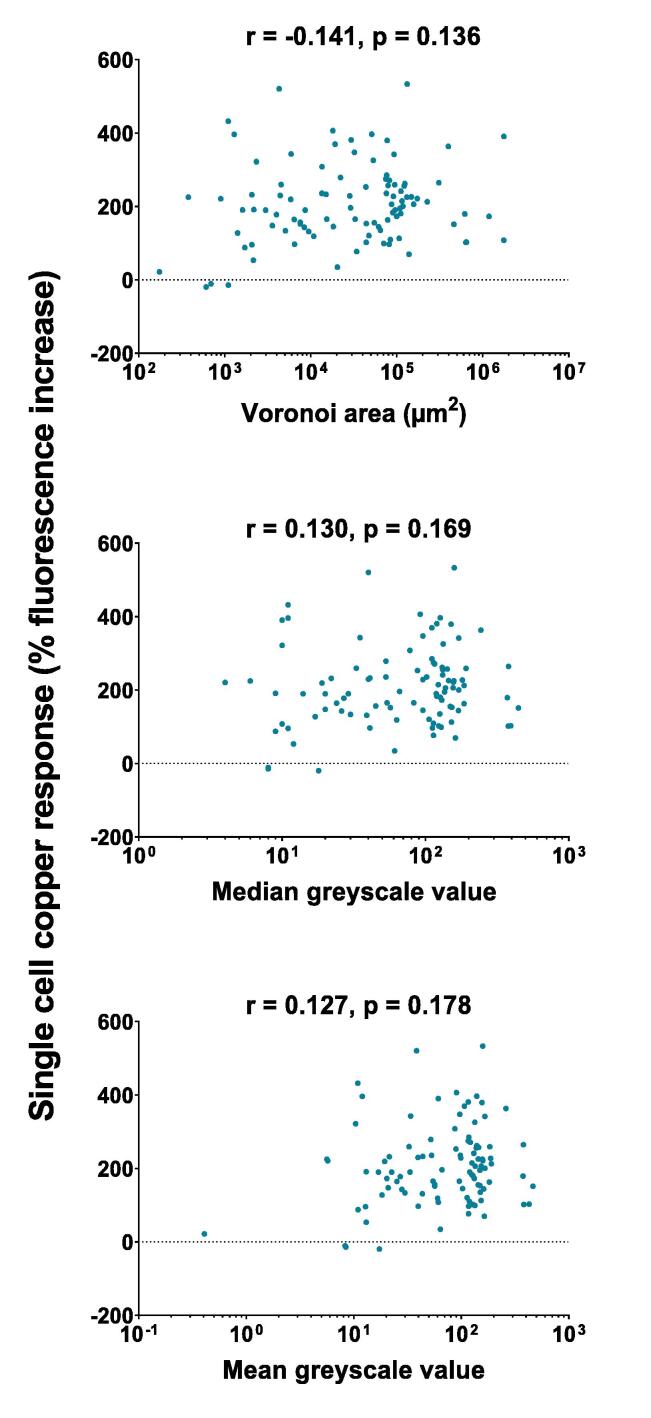
**The trialled spatial metrics do not predict single-cell copper response in soil micromodels.** Correlations between percentage increase in fluorescence (post- versus pre-copper) of cells expressing *pCUP1-*GFP and either Voronoi area (top panel), or median- (middle) or mean- (bottom) greyscale distance values of the Voronoi areas. Fluorescence was determined at 0 hr and 1 hr following exposure to 200 µM copper sulfate. n = 103; CV of single-cell fluorescence increase across the population = 53.7%.

Tools described above were applied to cells in microfluidic devices containing physical structure similar to that of soil particulates, i.e., soil micromodels, to explore relationships between that structure and yeast responses to copper. Introduction of cells to these devices resulted in approximately 100 cells per micromodel channel, with three identical replicate channels per micromodel (see Fig. 1). To expose cells to copper, YNB medium supplemented with 200 µm copper sulfate was flowed through the micromodels and their responses to the copper gauged by *pCUP1*-regulated GFP expression. Calculation of the percentage increases in single-cell fluorescence arising during copper exposure revealed that the responses were very heterogeneous, ranging from no detectable response in some cells to > 500% fluorescence increase in others (see y axis distributions, Fig. 4). It was hypothesised that cells within more confined spaces (e.g. smaller Voronoi area) would be more shielded from the flow of dissolved copper ions than cells in more open spaces. Spatial metrics analyses described above were applied to test this. However, no significant correlations were evident between the copper-response and Voronoi area of individual cells, or with weighted derivations (from greyscale mapping) of those areas (see Fig. 4 for Pearson’s correlation and p-values).

### 3.4 Modelling of fluid flow in soil micromodels and relationship with cellular responses to copper

**Fig. 5:**
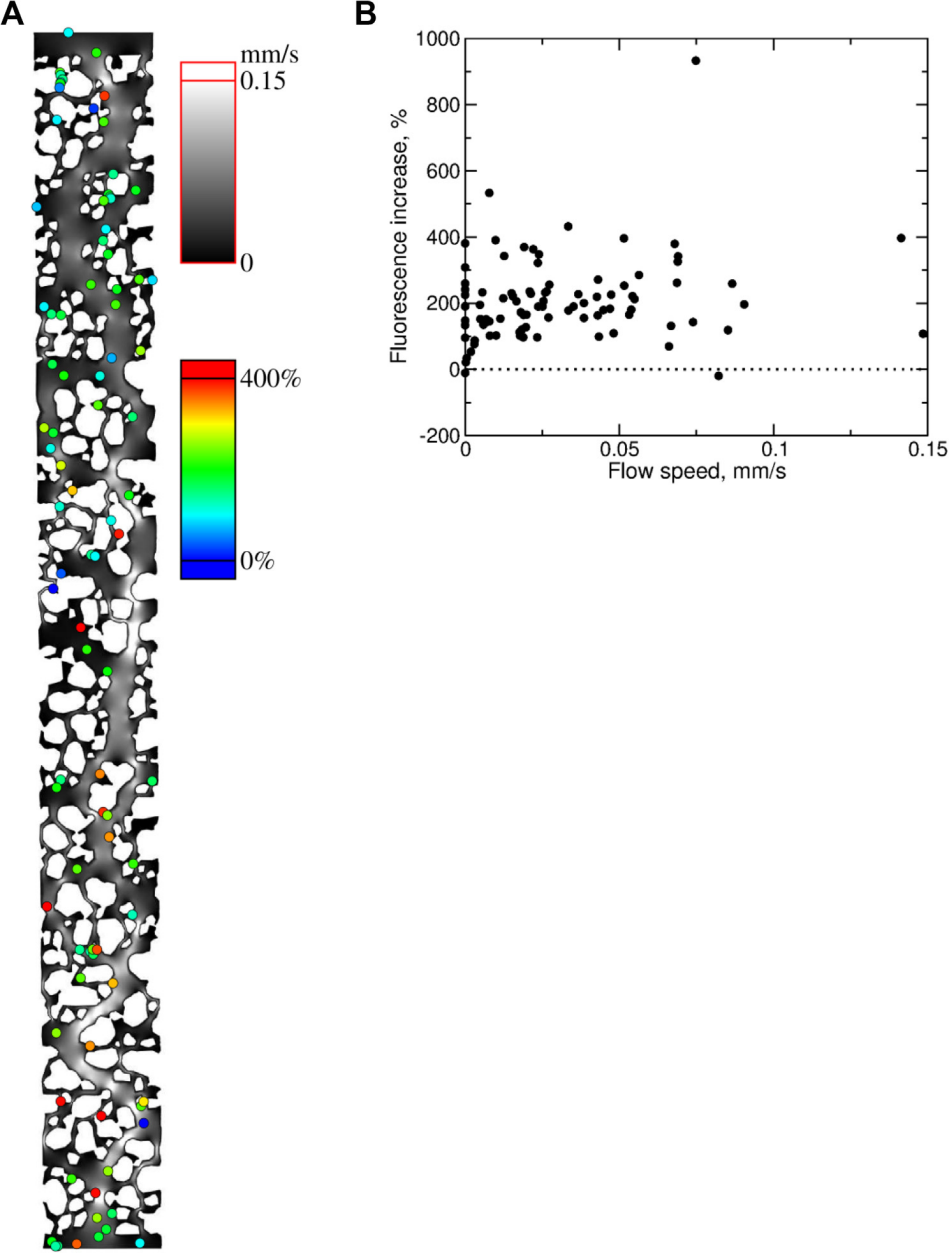
**Fluid flow simulation in soil micromodels and relationship with single-cell copper responses.** (A) Vertically averaged flow speeds (lighter shades of grey correspond to higher speeds; scale at top right) within the soil micromodel, calculated by solving numerically the set of equations (16), (12) (see 2.4.3), and experimentally measured % fluorescence increase in response to copper exposure of cells (coloured circles corresponding to coloured scale, right) expressing *pCUP1-*GFP. (B) The cells’ copper responses are plotted against vertically averaged flow speed at the cells’ different positions. (For interpretation of the references to colour in this figure legend, the reader is referred to the web version of this article.)

As the above spatial metrics did not predict cell responses in the soil micromodels, a computational fluid dynamics (CFD) approach was employed to model the fluid flow through these structures in order to correlate the rate of copper flow around single cells with their fluorescence responses. The system of equations (16), (12) (see 2.4.3) was solved with COMSOL Multiphysics software (COMSOL Ltd., Cambridge, UK) using the built-in Stokes flow solver, with the additional term proportional to **v** in Eq. (16) introduced as a body force. The vertically averaged flow speeds $\overset{-}{v}$ are shown in greyscale, with black representing regions of stagnation (i.e. no flow), and the fluorescence increase for individual cells is indicated using colour (Fig. 5**A)**. No correlation was apparent between the copper response (% fluorescence increase) of a cell and the flow speed around that cell (Fig. 5**B**). As described in the Methods, the ion flux towards a cell could be related to the average flow in a neighbourhood around the cell, rather than the (local) vertically averaged speed. However, it is unlikely that either case would give a correlation since Fig. 5**A** indicates that there are strongly copper-responsive cells in large regions with very little flow and, conversely, weakly responsive cells in large regions with significant flow. This suggests that factors other than copper flow rate alone are responsible for the differences in *pCUP1*-GFP expression between cells.

### 3.5 Examining yeast stress response in simplified structured environments

In the soil micromodels, the responses of individual cells to copper flow could not be described either by spatial metrics or fluid flow simulation. This may be in part due to the relatively complex structures within the model, but also that the structures themselves did not appear to introduce additional variation in cell–cell fluorescence response over-and-above that already seen in experiments for homogeneous environments of shake flasks (CV for cell fluorescence was 63.8% in shake flask experiments, compared to a CV of 53.7% in micromodel experiments; Fig. 2, Fig. 4). To seek to address these issues, a second experimental system was employed with a simplified but modifiable environmental structure. The CellASIC pad trap plate is a commercially available microfluidic device consisting of 4 polydimethylsiloxane (PDMS) chambers, each containing 104 barrier traps for retaining cells (see 2.5). By introducing 4-µm microspheres into the chamber, simple structured environments were created (Supp. Fig. 1**D**) alongside subsequently introduced yeast cells. First, preliminary experiments were conducted to determine whether the microspheres may alter overall fluid flow within traps (e.g. by blocking pores at the trap perimeter; see Supp. Fig. 1**D**). To track fluid flow, the fluorescent dye rhodamine 6G (R6G) was added and the labelled fluid was flowed at 8 psi into traps either containing or not microspheres. Microscopic examination of the dye movement over time revealed dye flow was decreased within the first ∼ 2 s of introduction in traps containing microspheres compared to those without microspheres (Supp. Fig. 2). However, after this initial difference in rate, the quantity of dye within each trap type was similar after approximately 4 s as the dye level in the microsphere-free traps had plateaued earlier. It was reasoned that the 2–4 s timescale of the initial difference was negligible relative to the 1 hr timescale of the copper-response assays. For the subsequent copper-response experiments, medium containing copper was introduced at 8 psi for 10 s (to give rapid equalization in traps with or without spheres) before continuing flow for 1 hr (at 2 psi) and analysis of cellular responses.

**Fig. 6:**
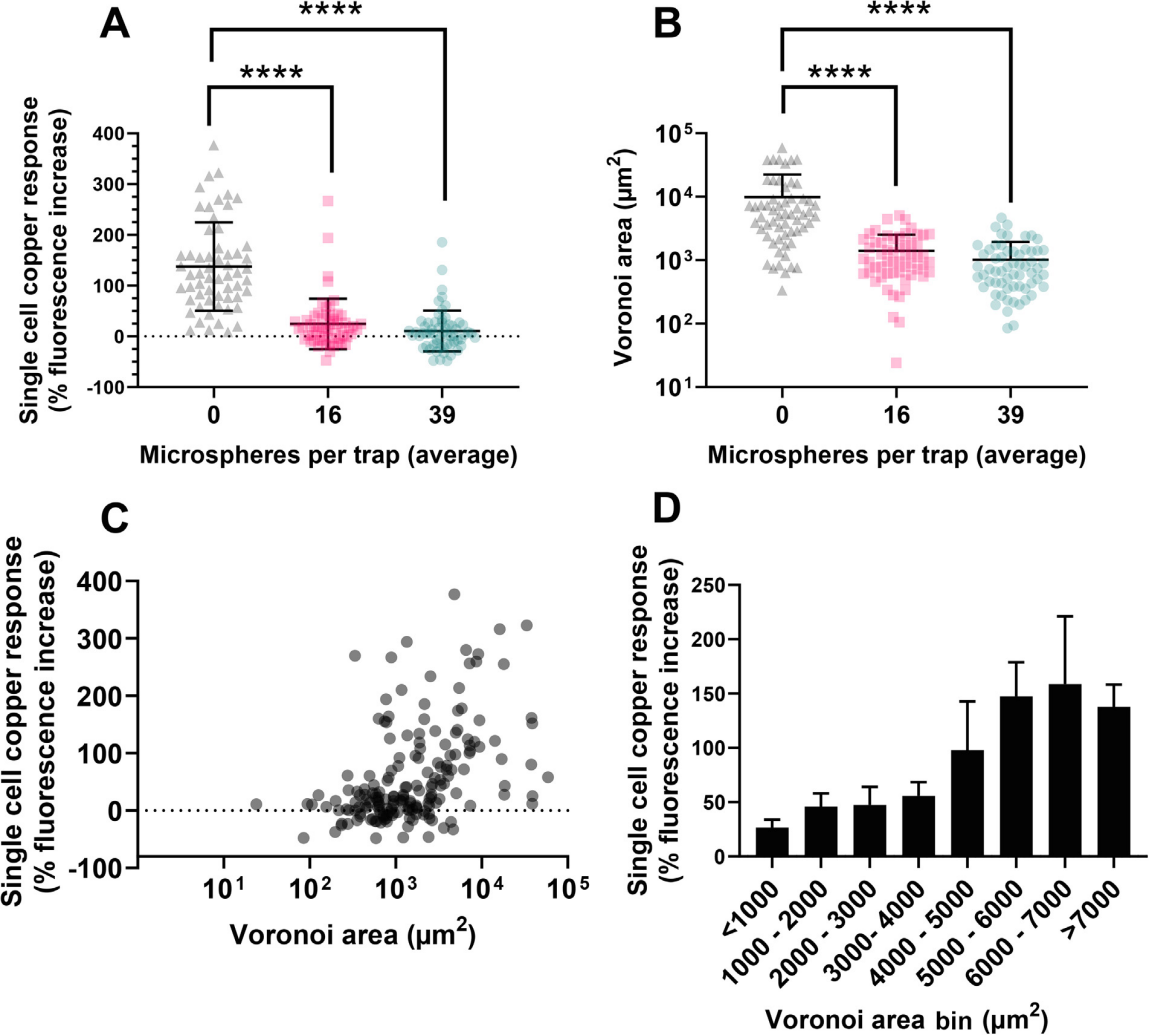
**Characterisation of decreased cellular response to copper inflow with increasing structure density in simplified structured environments.** (A) Responses to copper of *S. cerevisiae* cells expressing *pCUP1-*GFP in microfluidic chambers (CellASIC ONIX II) with an average of either 0, 16 (±5), or 39 (±8) microspheres per trap, after 1 hr exposure to 200 µM copper sulfate. Points represent individual cells, bars represent mean and standard deviation for 59, 58, or 62 cells (at the increasing microspheres per trap, respectively), with every cell being located in a separate structured environment (trap). CVs for single-cell % fluorescence increase were 63, 202, and 377% across cells in chambers with 0, 16, and 39 microspheres, respectively. (B) The Voronoi areas for individual cells determined across the same chambers as analysed in (A). Both (A) and (B) were analysed statistically by one-way ANOVA with Tukey’s multiple comparisons; ****p < 0.0001. (C) Correlation between single-cell Voronoi area and copper response (% fluorescence increase) of individual cells combined across the three structured and unstructured environments (Pearson’s r = 0.280, R^2^ = 0.079, p < 0.0001, n = 179). (D) Copper responses of cells binned in 1000 µm^2^ Voronoi-area intervals. Data shown are mean values ± SEM.

To investigate whether these micrometre-scale structured environments impacted cellular copper exposure, *S. cerevisiae* cells expressing *pCUP1-*GFP were introduced to traps and exposed to 200 µM copper sulfate for 1 hr under constant flow. Comparison of the mean fluorescence increase of cells in chambers either without added microspheres (unstructured) or with different quantities of microspheres (structured), revealed a decreased relative response in the structured environments (Fig. 6**A**) (one-way ANOVA with Tukey’s multiple comparisons, p < 0.0001 for both comparisons). There was no further significant difference in response between cells in chambers containing ∼ 16 or ∼ 39 microspheres per trap. The results suggested that introduction of structure into these environments suppressed cellular response (and, by inference, exposure) to copper.

Next, we investigated whether the spatial metric analysis presented earlier could help describe the differing responses of cells incubated with these simpler (compared to soil micromodels) microsphere-based structures in the microfluidic traps. There were significant, ∼7–12-fold reductions in the mean Voronoi areas (see 3.2) of cells in the structured environments (with added microspheres) compared to the unstructured (microsphere-free) control (Fig. 6**B**). This substantiated that cells within traps with more microspheres had reduced open space surrounding them. These spatial metrics showed a very similar trend as cellular response to copper inflow across the different structure densities (Fig. 6**A**). To interrogate this relationship further, the responses of each individual cell across all three conditions was assessed relative to its respective Voronoi area. This analysis at the single cell level showed a significant, positive correlation between a cell’s Voronoi area and its response to copper in the fluid flow (Pearson’s r = 0.237, p = 0.0014) (Fig. 6**C**). The largest absolute increases in fluorescence response of cells as their Voronoi increased occurred over areas ranging from 3000 − 6000 µm^2^ (Fig. 6**D**).

In a separate experiment omitting microspheres, a 50% reduction of the fluid flow rate of the copper-supplemented medium decreased the mean cellular response to copper by approximately 34% (Supp. Fig. 3). Given this and the facts that the presence of microspheres also decreased the copper response of cells (by 80 – 90%) (Fig. 6**A**) but did not substantially reduce flow into traps (except over the first few seconds) (Supp. Fig. 2), it was inferred that flow rates may be locally decreased near microsphere structures, and potentially by more than the 50% reduction trialled here. Accordingly, this would be expected to reduce the copper exposure of cells that are close to microspheres, and that is consistent with the fact that reduced Voronoi area was associated with reduced response (Fig. 6**C**). The data suggest that a cell’s Voronoi area becomes sufficient to describe, at least partly, its response to fluid-phase stressor in a simpler structured environment like that adopted here.

**Fig. 7:**
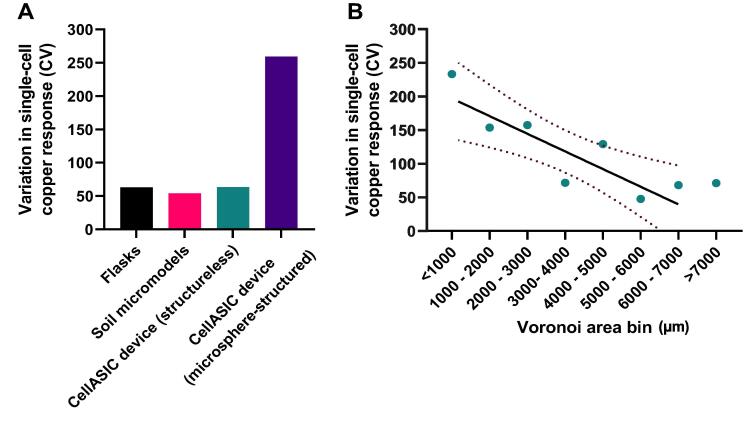
Cell-cell variation in fluorescence-response differs between experimental systems and correlates with cell Voronoi area in the microsphere system (CellASIC). (A) Variation in single-cell copper response (CV) in shake-flask and microfluidic experiments in response to copper exposure. Data derived from those of Fig. 2, Fig. 4, Fig. 6. (B) Correlation between Voronoi area of cells (binned as shown) and variation (CV) in their individual copper responses. Pearson’s r = -0.8458, R^2^ = 0.7154, p = 0.0081. Dotted lines represent 95% confidence intervals.

This rationale was further supported by comparing the cell–cell variation in fluorescence response that was evident within the two different experimental systems. Here, the microsphere structures (CellASIC system), but the not soil micromodels, increased the cell–cell variation (CV) above the level evident in flask-based experiments without structure (Fig. 7**A**). Countering the possibility that this reflected some other difference between the experimental systems than their level of structure, the CV of cellular fluorescence response was negatively correlated with cell Voronoi area in the system with microsphere structures (i.e., cells in larger spaces exhibited lower degrees of cell–cell variation) (Fig. 7**B**) and the CV became similar to that of the other systems when microspheres were omitted altogether to make it ‘structureless’ (Fig. 7**A**).

## 4 Discussion

This study examined the impact of micrometre-scale environmental structure on microbial stress response, as environments of such scale are common in the soil pore space [33] and environments where microbial growth is a concern (e.g. medical and hygiene settings) [30]. This was achieved with yeast cells expressing a stress (copper)-responsive reporter while incubated in microfluidic devices containing either structures that physically resembled soil particle sizes and shapes or small microspheres to create some simple physical structure. Spatial metrics were also developed and tested to quantify the proximities of cells to neighbouring environmental structures. In the soil micromodels, neither these metrics nor computational modelling of fluid flow were sufficient to predict relationships between the physical structure around a cell and its copper response. However, in the systems with simplified environmental structures, a significant relationship with spatial metrics did emerge, such that cells within more open spaces showed the greater induction of the *pCUP1-*GFP reporter during copper exposure.

Copper was selected as a suitable soluble stressor as yeast responses to copper are very well characterized and *CUP1,* which responds to high copper, is one of the most strongly inducible yeast genes so providing a convenient reporter of stressor sensing/exposure here [14], [17]. Accordingly, expression from a genomic insert containing GFP under control of the native *CUP1* promoter in *S. cerevisiae* SVY14 exhibited a strong, linear correlation with copper concentration over the range tested here (25–300 µM). This was sub-inhibitory to growth but sufficient to eliciting a strong transcriptional response. Regarding descriptors of relevant physical structure near cells, Voronoi tessellations have been used previously to describe distances between points in biological systems [4], [2]. These were adapted here to yeast cells and local environmental structure. In addition, an approach was developed to weight areas within a Voronoi tessellation, as nearby objects are likely to have the strongest effect on cell response (the tessellations have different shapes and this approach helped to resolve whether average or minimum/maximum distance from cells to objects was a factor in stress response).

It was hypothesised that cells within more enclosed spaces would be less exposed to fluid flow (containing dissolved copper) than cells within more open, exposed space, as it was anticipated that the flow may be obstructed and diverted by the structures. In soil micromodel experiments, differences in copper responsiveness of individual cells could not be correlated with differences in the spatial metric descriptors that were tested. Furthermore, modelling of the flow velocity around individual yeast cells also did not predict the cell–cell differences in response. This highlights the difficulties of disentangling interplay between environmental structure and microbial perturbation in even moderately complex systems, noting that the structure of natural soils is more complex again than that modelled here [6]. It is also important to bear in mind that substantial cell-to-cell phenotypic variation is prevalent even in uniform environments, as illustrated in the cell–cell variation seen in shake-flask based experiments here and in other studies [28], [11]. Therefore, only a certain fraction of cell-specific responses may be predictable by consideration of environmental structure.

Additional experiments were conducted in a different, simpler, microfluidics system consisting of arrays of traps to which microspheres were introduced, producing different structured environments. In contrast to the soil micromodel environments, a positive correlation between differences in copper responses of cells and relative size of their Voronoi areas in these simpler systems indicated that cells with microspheres in closer proximity were less exposed to the stressor and vice-versa. This difference between the two systems seemed to be reflected also in the fact that cell–cell variation in copper-response in soil micromodels was similar to that of cells exposed to copper in shake flasks (Fig. 7**A**), suggesting that the environmental structure in the micromodels did not add to the intrinsic biological heterogeneity of cellular response to copper [28]. In contrast, cell–cell response variation was much larger in experiments with added microspheres, coincident with emergence of the correlation with cell spatial metrics. One potential reason for the contrasting outcomes between the two microfluidics systems is that the traps in the CellASIC plates are arranged by design to minimise disruption of fluid flow from one trap to the next, whereas fluid flow in soil micromodels is dependent on upstream structures, as corroborated quantitatively by our modelling (Fig. 5). It is possible that introduced microspheres at trap openings alter the fluid flow into the trap, but evidence from dye tracking suggested this effect would be negligible here and would be significantly less than disruption by the much larger structures present in soil micromodels. Lastly we noted that, with decreasing Voronoi area of cells, the greatest absolute decreases in fluorescence-response occurred over areas spanning 6000 to 3000 µm^2^. This suggests a threshold of space over which copper response is weakened, which may help to inform future design of microfluidics devices for addressing similar research questions.

Whereas it can be difficult to identify relationships between complex structures and microbial-cell behaviour, as found here with the soil micromodels, the more deterministic relationship that emerged with the simpler microfluidic model offers the possibility that results could be interpreted in the context of any soluble agent influencing cell phenotype, such as nutrient or oxygen distribution within structured environments. However, this must be considered with caution, as the uptake of different substrates by cells varies in rate and extent alongside potential effects on cell growth [9], [7]. In addition, given the complexity of the environmental structures in and around which microorganisms can naturally reside, such as in soils or other porous media, caution is necessary when extrapolating these results to such environments. For example, many environments encompass semi-permeable structures, such as microbial extracellular polymeric substances (EPS) or biofilms that alter but not inhibit fluid movement [18]; or which support fluid flow in more than one direction, such as water filtering from aboveground and belowground in soils, which can alter fluid flow dynamics [27]. These additional complexities could be incorporated into future microfluidic designs, such as by incorporating semi-permeable hydrogels [6] or multiple flow inlets in the devices to simulate semipermeable structures and more complex flow dynamics [16].

## 5 Conclusions

Almost ubiquitously, the environments of microorganisms have three-dimensional structure and create heterogeneous distributions of the space in which microorganisms reside. Taking the outcomes with the two microfluidic designs used here, we establish that microscale structure can influence microbial stress sensing and response. However, detection of such a relationship is challenging even with the aid of computational modelling of fluid flow and in a controlled laboratory setup, in the absence of variability in other environmental factors such as nutrient and chemical distributions and seasonal/temporal transitions [21]. Future experiments could introduce some of these parameters to structured microfluidic devices, such as by fluctuation of stressor or nutrient exposure and/or capturing microbial adaptation in these environments over time — a consequence of living in any natural environment.

## Declaration of Competing Interest

The authors declare that they have no known competing financial interests or personal relationships that could have appeared to influence the work reported in this paper.
