# Supplementary material for: Application of microfluidic systems in modelling impacts of environmental structure on stress-sensing by individual microbial cells
Source: Comput Struct Biotechnol J. 2021 Dec 1;20:128–38. doi: 10.1016/j.csbj.2021.11.039 (PMC8689086; doi:10.1016/j.csbj.2021.11.039)
Supplement: Supplementary data 1 [file mmc1.docx]

**Supplementary Figures**


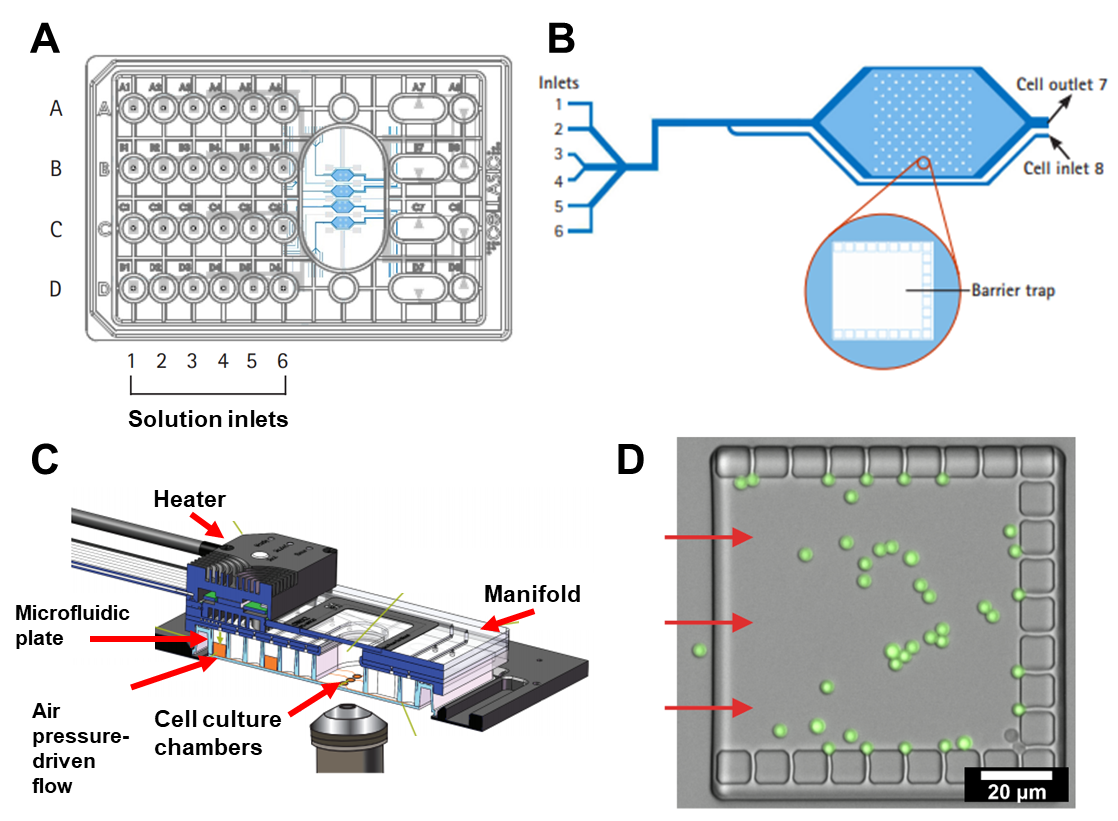


**Supplementary Figure 1- Overview of the CellASIC ONIX II microfluidic system and microfluidic plates**

Microfluidic plates (85.48 mm Width x 127.76 mm Length x 14.35 mm Height) (A) contain a series of inlets that can be filled with solutions to be introduced into one of four microfluidics chambers (blue, with one chamber illustrated in (B)). Note that in (A) letters A-D refer each to one chamber, allowing for up to 6 solution inlets per chamber. Each chamber contains 104 traps measuring 100x100 µm (B), designed to trap cells in place while still permitting fluid flow into and out of the traps. Fluid flow is introduced by pressure applied to each solution inlet via the manifold system (C), which seals the device and also regulates the temperature of the microfluidics plate. Illustrations (A-C) are adapted from the CellASIC ONIX II Microfluidics System User Guide (EMD Millipore). A microscopic image of a single trap (schematically highlighted in B) can be seen in (D) into which has been incorporated 4 µm microspheres (green spheres) and yeast cell inoculum (bottom right within trap). In (D), red arrows indicate the direction of fluid flow into the trap. Panels A, B, and C reproduced with permission from Merck KGaA, Darmstadt, Germany and/or its affiliates.

**Supplementary Figure 2- Flow rate differences in traps with and without microspheres**Rhodamine 6G (R6G) was introduced to CellASIC trap plate traps either containing microspheres or not. The fluid flow into individual traps was compared using R6G fluorescence after R6G inflow was initiated, with fluorescence values across the length of the trap averaged at every time point. The dotted line at Y = 16,383 represents the saturation value of the camera.

**Supplementary Figure 3- Reducing flow rate of copper-supplemented medium into the microfluidic traps reduces the cellular copper response**Cells in microfluidics traps were exposed to YNB supplemented with 200 µM copper nitrate at induced flow of either 2 or 1 PSI (corresponding to ~4 and 2 µL hr^-1^, respectively). ****, p < 0.0001 (two-sample *t-*test).
